# Body composition changes during cardiac rehabilitation and long-term cardiovascular outcomes in patients with coronary artery disease

**DOI:** 10.1016/j.ahjo.2026.100805

**Published:** 2026-05-20

**Authors:** Aarti Kumar, Christopher Van, Tara Shahrvini, Preethi Srikanthan, Tamara B. Horwich

**Affiliations:** a University of California, Los Angeles, Department of Internal Medicine, United States of America; b University of California, Los Angeles, United States of America; c University of California, Los Angeles, Department of Endocrinology, United States of America; d University of California, Los Angeles, Department of Cardiology, United States of America

**Keywords:** Body composition, Lean body mass, Body fat, Body mass index, Cardiac rehabilitation, Preventive cardiology

## Abstract

**Aims:**

This study assesses whether changes in body composition - lean body mass (LBM) and body fat % (BF%) - and body mass index (BMI), in patients undergoing cardiac rehabilitation (CR), are associated with long-term outcomes.

**Methods:**

In this cohort study of 1234 adults with coronary artery disease (CAD) who participated in CR from April 2012 to June 2024, bioelectric impedance analysis was used at baseline and after CR to assess body composition. Outcomes assessed included net adverse cardiovascular events (NACE) and all-cause mortality.

**Results:**

Subjects were followed for a mean of 5.9 years (SD 3.2). We found that those who had an increase in BF% during CR had a higher risk of NACE (adjusted HR 1.44, 95% CI: 1.10, 1.90) compared to those who had a decrease in BF%. There was also a trend towards decreased mortality in patients with a moderate increase in LBM (adjusted HR 0.49, 95% CI 0.23, 1.02). There were no associations between changes in body mass index and any outcomes. When the cohort was stratified by sex, both males and females had higher adjusted risk of NACE with BF% increase, but the finding was only statistically significant in males.

**Conclusion:**

An increase in BF% during CR was associated with increased NACE in patients with CAD. Overall, these findings suggest that interventions to address body composition change, particularly decreasing BF%, may be important in mitigating cardiovascular events in patients undergoing CR.

## Highlights

• An increase in body fat percentage during cardiac rehabilitation was associated with a higher risk of adverse outcomes.
• Interventions to reduce percentage body fat may reduce cardiovascular events in patients with CAD.
• Markers of body composition may be more informative to cardiovascular risk than the commonly used BMI.

## 1 Introduction

Obesity is a well-studied risk factor for coronary artery disease (CAD) [1], as it accelerates atherosclerosis through various mechanisms, including insulin resistance and inflammation [2]. However, while some studies have suggested that increased body mass index (BMI) is associated with increased risk of cardiovascular disease (CVD) [3], [4], other studies have demonstrated an obesity paradox in CVD, in which patients with a lower BMI have a worse prognosis [1], [5]. This may be due to the limitations of BMI as an accurate proxy for body fat (BF) [6], as the measure incorporates both adipose tissue and muscle mass. Thus, alternative measures of obesity that evaluate body composition, such as bioelectrical impedance analysis (BIA), may provide a better assessment of obesity since BIA measures both lean body mass (LBM) and body fat percentage (BF%) [7]. While some studies have demonstrated that muscle mass is associated with a decreased risk for CVD, others have noted adipose tissue is associated with increased risk [8], [9]. Further, it has been noted that adiposity has a preventive effect on cardiovascular mortality in women but not men at risk of CVD [10]. These divergent findings illustrate that the cardiovascular effect of components of body composition needs further investigation.

In patients with a cardiovascular event who subsequently participate in a cardiac rehabilitation (CR) program, studies have shown resulting weight loss with a decrease in body fat, but the long term effects of these changes have not been examined [11], [12], [13]. This study aimed to assess the associations between changes in BMI, BF%, and LBM in patients who have completed CR with long-term cardiovascular outcomes in CAD.

## 2 Methods

### 2.1 Study population and design

The study included a prospective cohort of 1234 patients ≥18 years old with CAD who participated in a CR program at the University of California, Los Angeles (UCLA) between April 1, 2012 and June 30, 2024 and completed BIA of body composition before and after CR; BIA was routinely utilized for body composition assessment. Indications for referral to CR included acute coronary syndrome, stable angina, percutaneous coronary intervention (PCI), and/or coronary artery bypass grafting (CABG). Patients with non-CAD diagnoses were excluded from analysis. Supplemental Fig. 1 further details exclusion criteria applied to arrive at the final cohort of 1234 patients.

Patients completed one of the two CR programs: traditional CR which totaled 24–36 one-hour exercise sessions over 8–12 weeks or intensive CR which totaled 72 exercise session hours (2 weekly 4-hour sessions) over 9 weeks with a similar exercise regimen as traditional CR but also incorporated nutritional education. For patients who underwent more than one course of CR of either traditional or intensive rehabilitation or both, only the first program course and the patient's baseline characteristics was included in the study. The study was approved by the Medical Institutional Review Board of UCLA.

### 2.2 Body composition measurements

Body composition was assessed by BIA using the InBody 230 from April 2012 to December 2015 and InBody 770 from January 2016 to June 2024 (Biospace Inc., Des Moines, IA). The InBody scale utilizes bioelectrical impedance to the flow of an electrical current (with lowest impedance in tissues with the highest water content), using a combination of low and high frequencies (InBody 230: 20 and 100 kHz; InBody 770: 1, 5, 50, 250, 500 and 1000 kHz), to estimate body composition - specifically lean mass indices - and uses a proprietary model to calculate fat mass indices. In accordance with manufacturer instructions, each patient remained in contact with bilateral scale hand and foot electrodes while several different frequencies of electrical currents were passed through the patient's arms, legs, and trunk. Measurements included total body water, dry lean mass, body fat mass, and segmental fat analysis of the right/left arms, right/left legs, and trunk. For the purposes of this study, the measures of interest were BF%, calculated from a fraction of total body fat mass over total body mass; LBM (kg), calculated from total body water plus dry lean mass; and BMI, calculated as weight (in kg) over height squared (in meters).

### 2.3 Outcomes

The primary outcome analyzed was net adverse clinical event (NACE), the composite outcome of all-cause mortality and major cardiovascular events (MACE), which encompassed: (1) acute coronary syndrome including ST- or non-ST-segment elevation myocardial infarction (MI) and unstable angina requiring hospitalization, (2) cardiac surgery including coronary revascularization with PCI and/or CABG, valve repair, or heart transplantation (3) acute decompensated heart failure requiring hospitalization, or (4) stroke. The secondary outcome was all-cause mortality. Covariates included age, sex, race, smoking history, alcohol history, diabetes mellitus, hypertension and history of MI. All variables were gathered from chart review and CR intake forms.

### 2.4 Statistical analysis

Descriptive baseline patient characteristics were reported as frequencies with percentages for categorical data and means and standard deviations (SD) for continuous data. Change in BMI was calculated as a patient's BMI before rehabilitation subtracted from their BMI after rehabilitation divided by their pre-rehabilitation BMI multiplied by 100 to calculate a percentage. Changes in BF% and LBM were similarly calculated. The cohort was also stratified by quartile of each body composition group (Table 1). The *p*-values for comparing body composition measures before and after CR were computed with the paired *t*-test. The association between quartiles of body composition variables versus time to NACE was assessed using the multivariable Cox proportional hazards. All patients who died had a preceding MACE; thus, MACE and mortality were not competing risks and competing risk analysis was not performed. Because the association between body composition measures and the hazard of NACE may not be linear across the range of values, we used categorical rather than continuous measures for body composition. MACE and mortality were also evaluated as separate outcomes. Models were adjusted for age, gender, race, smoking history, alcohol history, diabetes, hypertension, and a history of MI; analyses examining change in BF% also adjusted for LBM and vice versa as done in previous studies [14] because BF% and LBM may have separate effects on the outcomes. In addition to comparing quartiles, subjects who had an overall increase in each of the body composition measure variables after CR were compared to those with a decrease in that variable after CR. There was a significant interaction between body composition measures and sex with NACE, so stratified analyses were performed by sex in addition to analyses with the total cohort. For all tests, a *p*-value <0.05 was considered statistically significant. All analyses were performed using Stata 18.

**Table 1:** Overview of BMI, BF%, and LBM changes by quartile in study cohort (*n* = 1234).

	Quartile 1	Quartile 2	Quartile 3	Quartile 4
BMI Change (%) (median, range)	−4.6 (−15.4, −2.8)	−1.7 (−2.8, −0.8)	0 (−0.8, 0.9)	2.4 (0.9, 14.4)
BF% Change (%) (median, range)	−12.2 (−54.8, −8.2)	−5.3 (−8.1, −3.0)	−0.7 (−3.0, 1.8)	5.6 (1.8, 41.1)
LBM Change (%) (median, range)	−3.1 (−22.1, −1.8)	−0.8 (−1.8, 0.1)	1.1 (0.1, 2.2)	3.5 (2.2, 15.3)
BMI Change (%) (mean, SD)	−5.0 (1.87)	−1.7 (0.6)	0.03 (0.4)	2.9 (2.0)
BF% Change (%) (mean, SD)	−14.2 (6.3)	−5.4 (1.5)	−0.8 (1.4)	7.8 (6.9)
LBM Change (%) (mean, SD)	−3.7 (2.1)	−0.8 (0.6)	1.1 (0.6)	4.0 (1.6)

BF: body fat, BMI: body mass index, LBM: lean body mass. Change was calculated as a patient's BMI before rehabilitation subtracted from their BMI after rehabilitation divided by their pre-rehabilitation BMI multiplied by 100 to calculate a percentage. Changes in BF% and LBM were similarly calculated.

## 3 Results

### 3.1 Baseline characteristics

The study cohort consisted of 1234 participants. Baseline characteristics are summarized in Table 2; a majority of participants were white males in their mid-60s. Those with a higher BMI at baseline tended to be younger and had a higher prevalence of diabetes and hypertension. Those with a higher baseline BF% were older, more often female, had a greater proportion who never used alcohol, and greater prevalence of diabetes and hypertension. In contrast, those with a higher LBM at baseline were younger, more often male, more often white, and had a greater proportion with current or former alcohol use (Supplemental Table 1).

**Table 2:** Baseline characteristics of study cohort (*n* = 1234).

Demographics	
Age (years), mean (SD)	66.5 (11.5)
Male sex, n (%)	928 (75.2%)
Race	
White, n (%)	742 (60.1%)
Asian, n (%)	91 (7.4%)
Black, n (%)	73 (5.9%)
Hispanic/Latino, n (%)	52 (4.2%)
Middle Eastern, n (%)	26 (2.1%)
Multiethnic, n (%)	11 (0.9%)
Alaska Native/American Indian, n (%)	8 (0.7%)
Native Hawaiian/Pacific Islander, n (%)	7 (0.6%)
Other, n (%)	42 (3.4%)
Prefer not to disclose, n (%)	182 (14.8%)
Ever alcohol, n (%)	596 (48.3%)
Ever smoker, n (%)	423 (34.3%)
Diabetes, n (%)	323 (26.2%)
Hypertension, n (%)	931 (75.5%)
History of myocardial infarction, n (%)	705 (57.1%)


Body composition	
BMI, mean (SD)	27.1 (4.8)
BF%, mean (SD)	27.7 (9.3)
LBM (kg), mean (SD)	57.4 (12.1)
Underweight (BMI <18.5) n (%)	13 (1.1%)
Normal weight (BMI 18.5–24.9), n (%)	438 (35.5%)
Overweight (BMI 25–29.9), n (%)	492 (39.9%)
Obesity (BMI > 30), n (%)	291 (23.6%)

BF: body fat, BMI: body mass index, LBM: lean body mass.

### 3.2 Changes in body composition during CR

Subjects were followed for a mean of 5.9 years (SD 3.2) after completion of CR. There was a decrease in mean BMI (mean 27.1, SD 4.8 compared to mean: 26.8, SD 4.7) and BF% (mean 27.7, SD 9.3 compared to mean 26.6, SD 9.3) after rehabilitation, which were both statistically significant by paired *t*-tests (*p* < 0.001), while there was a non-significant (*p* = 0.55) increase in LBM (mean 57.4, SD 12.1 compared to mean 57.5, SD 12.0). In comparing baseline characteristics by changes in BMI, BF%, and LBM before and after CR by quartiles, those with an increase in BMI and BF% had a higher prevalence of diabetes and tended to be older (Table 3).

**Table 3:** Baseline characteristics of study cohort by quartiles of changes in BMI, BF%, and LBM (n = 1234).

	BMI Change Q1 [−15.4, −2.8)	BMI Change Q2 [−2.8, −0.8)	BMI Change Q3 [−0.8, 0.9)	BMI Change Q4 [0.9, 14.4]	Total	P-value
Age (years), mean (SD)	63.6 (11.6)	68 (11.5)	67.1 (10.8)	67.1 (11.7)	66.5 (11.5)	**<0.001**
Male sex, n (%)	232 (75.1%)	234 (76.0%)	232 (75.1%)	230 (74.7%)	928 (75.2%)	0.986
White race, n (%)	206 (66.7%)	181 (58.8%)	179 (57.9%)	176 (57.1%)	742 (60.1%)	0.055
Ever alcohol, n (%)	149 (48.2%)	146 (47.4%)	149 (48.2%)	152 (49.4%)	596 (48.3%)	0.972
Ever smoker, n (%)	105 (34.0%)	113 (36.7%)	100 (32.4%)	105 (34.1%)	423 (34.3%)	0.728
Diabetes, n (%)	64 (20.7%)	73 (23.7%)	81 (26.1%)	105 (34.1%)	323 (26.2%)	**0.001**
Hypertension, n (%)	232 (75.1%)	235 (76.3%)	219 (70.9%)	245 (80.0%)	931 (75.5%)	0.094
History of myocardial infarction, n (%)	180 (58.3%)	176 (57.1%)	185 (59.9%)	165 (53.6%)	706 (57.2%)	0.442


	BF% Change Q1 [−54.8, −8.2)	BF% Change Q2 [−8.2, −3.0)	BF% Change Q3 [−3.0, 1.8)	BF% Change Q4 (1.8, 41.1]	Total	P-value
Age (years), mean (SD)	63.7 (12.1)	67.7 (11.4)	67.4 (10.2)	67.2 (11.7)	66.5 (11.5)	**<0.001**
Male sex, n (%)	261 (84.5%)	222 (72.1%)	211 (68.3%)	234 (76.0%)	928 (75.2%)	**<0.001**
White race, n (%)	194 (62.8%)	181 (58.8%)	179 (57.9%)	188 (61.0%)	742 (60.1%)	0.599
Ever alcohol, n (%)	159 (51.5%)	152 (49.4%)	148 (47.9%)	137 (44.5%)	596 (48.3%)	0.364
Ever smoker, n (%)	99 (32.0%)	116 (37.7%)	100 (32.4%)	108 (35.1%)	423 (34.3%)	0.420
Diabetes, n (%)	49 (15.9%)	76 (24.7%)	103 (33.3%)	95 (30.8%)	323 (26.2%)	**<0.001**
Hypertension, n (%)	225 (72.8%)	230 (74.7%)	247 (79.9%)	229 (74.4%)	931 (75.5%)	0.177
History of myocardial infarction, n (%)	192 (62.1%)	178 (57.8%)	168 (54.4%)	168 (54.6%)	706 (57.2%)	0.169


	LBM Change Q1 [−22.1, −1.8)	LBM Change Q2 [−1.8, 0.1)	LBM Change Q3 [0.1, 2.2)	LBM Change Q4 [2.2, 15.3]	Total	P-value
Age (years), mean (SD)	66.3 (11.6)	65.9 (11.6)	65.6 (11.6)	68.1 (11.0)	66.5 (11.5)	**0.028**
Male sex, n (%)	225 (72.8%)	236 (76.6%)	249 (80.6%)	218 (70.8%)	928 (75.2%)	**0.024**
White race, n (%)	198 (64.1%)	191 (62.0%)	168 (54.4%)	185 (60.1%)	742 (60.1%)	0.082
Ever alcohol, n (%)	152 (49.2%)	150 (48.7%)	153 (49.5%)	141 (45.8%)	596 (48.3%)	0.781
Ever smoker, n (%)	110 (35.6%)	112 (36.4%)	96 (31.1%)	105 (34.1%)	423 (34.3%)	0.520
Diabetes, n (%)	83 (26.9%)	69 (22.4%)	78 (25.2%)	93 (30.2%)	323 (26.2%)	0.169
Hypertension, n (%)	222 (71.8%)	228 (74.0%)	235 (76.1%)	246 (79.9%)	931 (75.5%)	0.119
History of myocardial infarction, n (%)	162 (52.4%)	175 (56.8%)	189 (61.2%)	180 (58.4%)	706 (57.2%)	0.167

BF: body fat, BMI: body mass index, LBM: lean body mass. Ranges for each quartile are noted above. Anova was used for continuous measures and Fisher's exact was used for categorical variables. Bolded text signifies p<0.05.

### 3.3 Association between body composition changes and clinical outcomes

Over the follow-up time, 7.5% of the cohort died at a median of 4 years. In terms of the other MACE outcomes of interest, the incidence of cardiac surgery was most common (8.4%), followed by MI (5.6%), heart failure exacerbation requiring hospitalization (4.9%), and stroke (2.3%) (Supplemental Table 2).

**Fig. 1:**
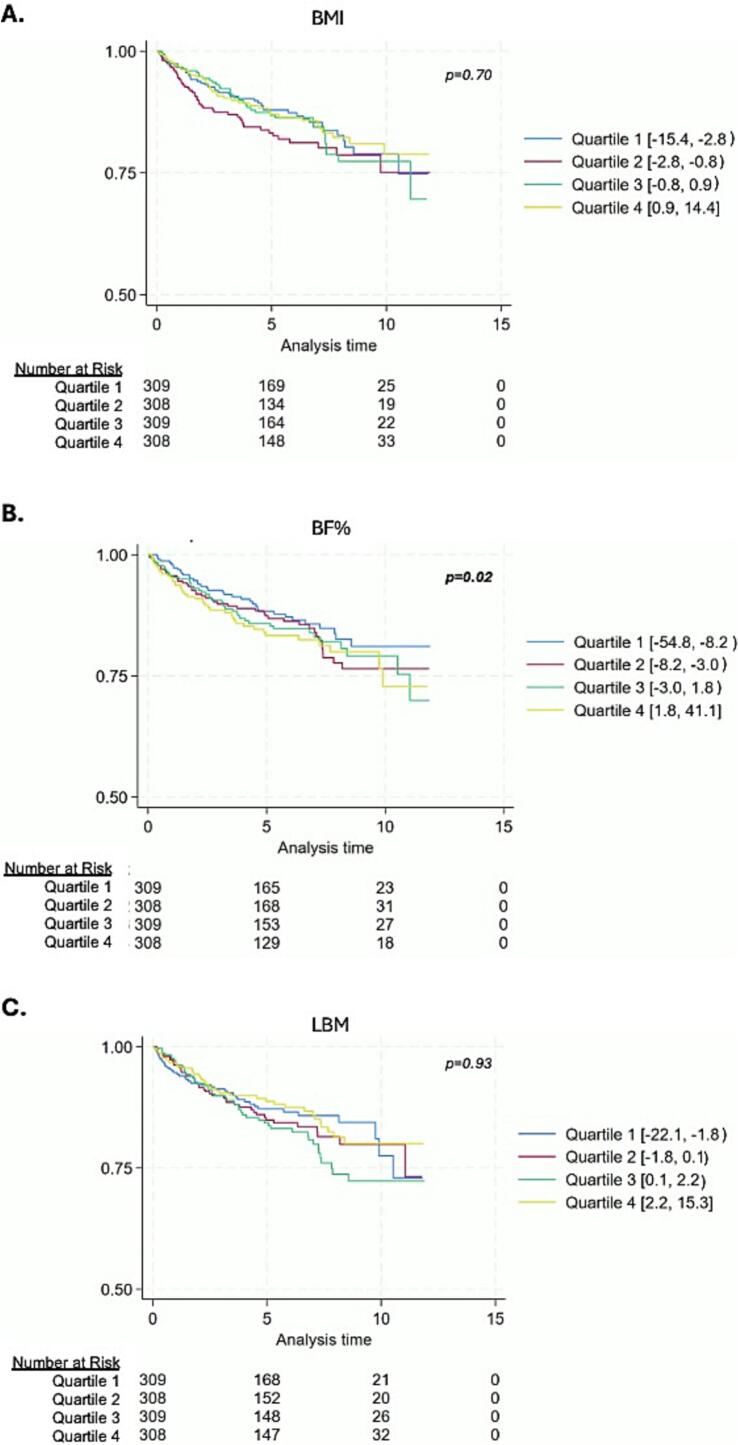
Kaplan-Meier survival curves by quartiles of changes in (A) BMI, (B) BF%, and (C) LBM. Log-rank tests were statistically significant for BF% (*p* = 0.02), but not BMI (*p* = 0.70) or LBM (*p* = 0.93).

In unadjusted Kaplan-Meier survival curves by quartile of percentage change in BF%, LBM, and BMI, there was a notable trend observed, with increased BF% corresponding to greater risk of NACE (log rank *p*-value = 0.02) whereas there were no significant associations with BMI (log rank p-value = 0.70) or LBM (log rank p-value = 0.93) (Fig. 1). In Cox regressions to assess whether changes in body composition measures by quartiles were associated with long-term cardiovascular outcomes, the fourth quartile for BF% change (range +1.8% to +41.4%) compared to the first quartile (range −54.8% to −8.2%) was significantly associated with increased risk of the composite outcome of NACE (HR 1.66, 95% CI 1.18, 2.34) as well as mortality alone (HR 2.20, 95% CI 1.26, 3.85), albeit these associations were attenuated and no longer significant after adjustment for covariates (Table 4). The third quartile of LBM (range +0.1% to +2.2%) was associated with lower risk of mortality alone compared to the first quartile (range −22.1% to −1.8%) in unadjusted analyses (HR 0.36, 95% CI 0.18, 0.71), which again was no longer significant when adjusted for covariates. There were no significant associations with BMI changes (Table 4).

**Table 4:** Associations between changes in BMI, BF%, and LBM by quartiles with long-term cardiovascular outcomes.

	MACE only		Mortality only		NACE	
	Unadjusted HR	Adjusted HR^a^	Unadjusted HR	Adjusted HR^a^	Unadjusted HR	Adjusted HR^a^
BMI Change (%)						
Quartile 1 [−15.4, −2.8)	**1 (Ref)**	**1 (Ref)**	**1 (Ref)**	**1 (Ref)**	**1 (Ref)**	**1 (Ref)**
Quartile 2 [−2.8, −0.8)	1.32 (0.87, 1.99)	1.23 (0.81, 1.87)	1.01 (0.56, 1.80)	0.65 (0.35, 1.18)	1.19 (0.85, 1.66)	1.00 (0.71, 1.41)
Quartile 3 [−0.8, 0.9)	1.06 (0.69, 1.62)	1.00 (0.65, 1.54)	0.87 (0.48, 1.56)	0.62 (0.34, 1.14)	0.99 (0.70, 1.39)	0.86 (0.60, 1.21)
Quartile 4 [0.9, 14.4]	0.97 (0.63, 1.50)	0.91 (0.59, 1.41)	1.19 (0.69, 2.05)	0.82 (0.47, 1.43)	1.04 (0.74, 1.46)	0.88 (0.62, 1.24)

BF% Change (%)						
Quartile 1 [−54.8, −8.2)	**1 (Ref)**	**1 (Ref)**	**1 (Ref)**	**1 (Ref)**	**1 (Ref)**	**1 (Ref)**
Quartile 2 [−8.1, −3.0)	1.25 (0.81, 1.93)	1.16 (0.74, 1.79)	0.95 (0.50, 1.81)	0.60 (0.31, 1.15)	1.13 (0.79, 1.62)	0.93 (0.65, 1.34)
Quartile 3 [−3.0, 1.8)	1.26 (0.81, 1.94)	1.13 (0.72, 1.77)	1.15 (0.62, 2.14)	0.94 (0.49, 1.81)	1.20 (0.84, 1.72)	1.01 (0.70, 1.46)
Quartile 4 [1.8, 41.1]	1.39 (0.90, 2.15)	1.36 (0.85, 2.17)	**2.20 (1.26, 3.85)**^b^	1.48 (0.77, 2.86)	**1.66 (1.18, 2.34)**	1.43 (0.98, 2.09)

LBM Change (%)						
Quartile 1 [−22.1, −1.8)	**1 (Ref)**	**1 (Ref)**	**1 (Ref)**	**1 (Ref)**	**1 (Ref)**	**1 (Ref)**
Quartile 2 [−1.8, 0.1)	1.23 (0.73, 1.73)	1.17 (0.76, 1.81)	0.72 (0.42, 1.24)	0.87 (0.50, 1.53)	0.93 (0.67, 1.31)	1.03 (0.73, 1.45)
Quartile 3 [0.1, 2.2)	1.32 (0.87, 1.99)	1.37 (0.89, 2.13)	**0.36 (0.18, 0.71)**	0.49 (0.23, 1.02)	0.89 (0.64, 1.26)	1.02 (0.71, 1.47)
Quartile 4 [2.2, 15.3]	0.97 (0.62, 1.51)	1.00 (0.62, 1.62)	0.91 (0.55, 1.51)	0.98 (0.53, 1.80)	0.93 (0.66, 1.30)	0.99 (0.68, 1.43)

BF: body fat, BMI: body mass index, HR: hazards ratio, LBM: lean body mass, MACE: major adverse cardiovascular events, NACE: net adverse cardiovascular events.

a Adjusted for age, gender, race, smoking history, alcohol history, diabetes, hypertension, and history of myocardial infarction. Models for BF% are also adjusted for LBM. Models for LBM are also adjusted for BF%.

b Bolded estimates indicate those that were statistically significant with p < 0.05.

In analyses examining subjects who had an increase in BF%, LBM, or BMI after CR compared to those with the same level or decrease in these measures after CR, those with increased BF% had a higher risk for NACE (adjusted HR 1.44, 95% CI 1.10, 1.90) as well as mortality alone (adjusted HR 1.75, 95% CI 1.09, 2.80) even after adjustment for covariates. There were no significant associations with BMI or LBM (Table 5).

**Table 5:** Associations between increase vs. decrease in BMI, BF%, and LBM with long-term cardiovascular outcomes.

	MACE only		Mortality only		NACE	
	Unadjusted HR	Adjusted HR^a^	Unadjusted HR	Adjusted HR^a^	Unadjusted HR	Adjusted HR^a^
BMI Stable or Decreased	**1 (Ref)**	**1 (Ref)**	**1 (Ref)**	**1 (Ref)**	**1 (Ref)**	**1 (Ref)**
BMI Increased	1.03 (0.75, 1.41)	1.00 (0.73, 1.37)	1.12 (0.74, 1.71)	0.97 (0.64, 1.49)	1.06 (0.82, 1.36)	0.99 (0.77, 1.28)
BF% Stable or Decreased	**1 (Ref)**	**1 (Ref)**	**1 (Ref)**	**1 (Ref)**	**1 (Ref)**	**1 (Ref)**
BF% Increased	1.18 (0.86, 1.63)	1.24 (0.88, 1.75)	**2.10 (1.40, 3.16)**^b^	**1.75 (1.09, 2.80)**	**1.47 (1.15, 1.89)**	**1.44 (1.10, 1.90)**
LBM Stable or Decreased	**1 (Ref)**	**1 (Ref)**	**1 (Ref)**	**1 (Ref)**	**1 (Ref)**	**1 (Ref)**
LBM Increased	1.11 (0.83, 1.50)	1.14 (0.83, 1.58)	0.71 (0.47, 1.08)	0.76 (0.47, 1.22)	0.95 (0.75, 1.22)	1.01 (0.77, 1.32)

BF: body fat, BMI: body mass index, HR: hazards ratio, LBM: lean body mass, MACE: major adverse cardiovascular events, NACE: net adverse cardiovascular events.

a Adjusted for age, gender, race, smoking history, alcohol history, diabetes, hypertension, and history of myocardial infarction. Models for BF% are also adjusted for LBM. Models for LBM are also adjusted for BF%.

b Bolded estimates indicate those that were statistically significant with *p* < 0.05.

**Fig. 2:**
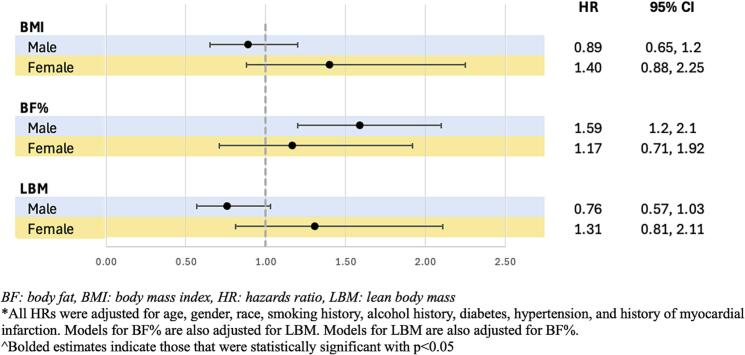
Associations between increased compared to decreased BMI, BF%, and LBM after CR with NACE stratified by gender.

Given there were significant interactions between lean body mass and gender (p for interaction = 0.04), stratified analyses were performed to assess for differences in associations between increases in body composition measures by sex with NACE. While there was a trend towards increased LBM being associated with increased NACE in females (unadjusted HR 1.51, CI 0.95–2.40, adjusted HR 1.31, CI 0.81–2.11) but decreased risk of NACE in males (unadjusted HR 0.80, CI 0.60–1.07, adjusted HR 0.76, CI 0.57–1.03), these associations were not statistically significant. In addition, the higher risk associated with increased BF% after multivariable adjustment was observed in both sexes, but only statistically significant in males (unadjusted HR 1.68, CI 1.25–2.25; adjusted HR 1.59, CI 1.20–2.10) but not females (unadjusted HR 1.03, CI 0.64–1.66, adjusted HR 1.17, CI 0.71–1.92) (Fig. 2).

## 4 Discussion

In this prospective cohort study examining body composition changes in patients who have completed CR with long-term cardiovascular outcomes, an increase in BF% was associated with a higher risk of adverse outcomes, whereas there were no consistent associations with BMI or LBM.

There were no significant associations between BMI and NACE in our study. Though BMI is the most commonly utilized measurement in obesity-related research as well as in clinical practice, it may not be the most appropriate measure to understand the relation between obesity and cardiometabolic risk. Previous studies examining associations between BMI and cardiovascular disease have shown varying findings. While higher BMI is associated with increased risk of incident CVD [3], [4], in patients with established cardiovascular disease, an obesity paradox has been noted, such that patients with a lower BMI have a worse prognosis [1], [5], suggesting that other components of body composition are also important to consider, as demonstrated in the current study.

Other groups have similarly demonstrated that BF%, rather than BMI, is associated with cardiovascular outcomes [15], [16], [17], likely due to limitations in the ability of BMI to estimate BF and due to variations in the association of BMI with BF% by with sex, age, and race/ethnicity [6], [18], [19], [20]. In stratified analyses by gender, we found that while increased body fat was associated with cardiovascular disease in men, this association was not significant in women, though there was a similar trend. It is important to note that the cohort consisted of more men (75%) than women, which could explain these results. Interestingly, in a previous study in healthy women, high fat, regardless of muscle mass level, was associated with lower CVD mortality risk [10]. Another study showed that visceral adipose tissue in women was more strongly associated with cardiometabolic risk markers, while lower extremity fat was relatively protective compared to men, suggesting that the importance of the quantity, quality, and location of fat may differ by sex [21]. However, it is unknown whether the increase in BF% itself is driving risk or whether the increase in BF% is a marker of other physiologic markers or lifestyle changes that affect risk.

Several studies have shown a protective effect of LBM on CVD [9], [22], [23]. In a previous study from this group in a similar cohort, in sex-stratified analyses, the moderate compared to low LBM at baseline was associated with decreased risk of all-cause mortality in women but not men [24]. In the present study, in the overall cohort, there was a decreased risk of mortality in patients in the third LBM quartile (those with median increase in LBM by 1.1%) compared to the first quartile (adjusted HR 0.49, 95% CI 0.23, 1.02), though this was non-significant after adjusting for covariates. In sex-stratified analyses, this trend held true in men but not women. However, it should be noted that the confidence interval for the overall cohort was wide and there were only small changes in LBM before and after cardiac rehabilitation. In addition, there were fewer women in the cohort compared to men, which could explain the different results by sex.

This study has several strengths. Subjects were followed for several years to accrue data on long-term cardiovascular outcomes. Bioelectric impedance testing was collected both before and after CR. Furthermore, BIA is a non-invasive, feasible, and low-cost method to assess body composition that can be scaled widely.

There are some limitations to note. Data gathered was limited to chart review from our institution. Therefore, it is possible that patients experienced adverse outcomes that were not reflected in our analysis if they presented at an outside hospital, where records were not transferred or linked, or if they did not seek medical care all together. In addition, the majority of the cohort was white males in their 60s, so more nuanced associations related to demographic differences may not be accurately captured. Further, while we focused on BMI, BF%, and LBM in this study, there are other measures gathered from BIA that would be beneficial to assess in future studies, such as segmental fat analysis or visceral fat volume. This may be important given that there is a difference in the metabolic significance of different adipose tissue depots, with thigh and hip fat having a positive metabolic effect compared to the detrimental cardiometabolic effect of visceral adipose tissue depots [25], [26], [27], [28]. We acknowledge the changes in BF% and LBM over time are small. There is chance that the cohort is biased, in that patients who complete both the CR program and the follow-up body composition assessments and thus are included in the study may be inherently more motivated to improve their health compared to those who drop out. Furthermore, additional studies are needed to confirm whether the associations between body composition and their association with outcomes are sex-specific.

Overall, our study of change in BF%, LBM, and BMI in a group of patients with CVD who completed CR suggests that BMI change over time is a poor indicator of cardiovascular outcomes. BF% elevation had a significant association with adverse cardiovascular outcomes, particularly in males, while LBM change showed a non-significant trend towards a protective effect with cardiovascular outcomes in men. This is suggestive that interventions to address body composition change, particularly decreasing BF% and increasing LBM, may be important to reduce cardiovascular outcomes in these cardiovascular patients. Additional research on body composition changes and long-term outcomes in patients with CAD are needed to guide appropriate lifestyle recommendations in this population.

## CRediT authorship contribution statement

**Aarti Kumar:** Writing – review & editing, Writing – original draft, Methodology, Investigation, Formal analysis, Data curation, Conceptualization. **Christopher Van:** Writing – review & editing, Data curation. **Tara Shahrvini:** Writing – review & editing, Data curation. **Preethi Srikanthan:** Writing – review & editing, Supervision, Project administration, Methodology, Investigation, Data curation, Conceptualization. **Tamara B. Horwich:** Writing – review & editing, Supervision, Project administration, Methodology, Investigation, Data curation.

## Ethical statement

Verbal informed consent to participate was obtained from all participants in the study. The study was approved by the Medical Institutional Review Board of UCLA.

## Funding

None.

## Declaration of competing interest

The authors declare that they have no known competing financial interests or personal relationships that could have appeared to influence the work reported in this paper.
