# Supplementary material for: Body composition changes during cardiac rehabilitation and long-term cardiovascular outcomes in patients with coronary artery disease
Source: Am Heart J Plus. 2026 May 20;67:100805. doi: 10.1016/j.ahjo.2026.100805 (PMC13226217; doi:10.1016/j.ahjo.2026.100805)
Supplement: Supplementary file 1 — Supplementary material [file mmc1.docx]

Supplemental Figure 1.

Outliers based on subjects with body composition changes that were outside 1.5*IQR

*n=56*

Patients with subsequent CR sessions

*5 patients with 3 CR sessions, 49 patients with 2 CR sessions*

Missing body composition metrics

*n=615*

Non-CAD diagnosis codes

*n=156*

Supplemental Table 1. Baseline characteristics of study cohort by quartiles of initial BMI, BF%, and LBM (n=1234).

|  | Baseline BMI Q1 [15.8, 23.7) | Baseline BMI Q2 [23.7, 26.5) | Baseline BMI Q3 [26.5, 29.7) | Baseline BMI Q4 [29.7, 53.9) | Total | P-value |
| --- | --- | --- | --- | --- | --- | --- |
| Age (years), mean (SD) | 68.8 (12.3) | 67.3 (10.5) | 65.0 (11.7) | 64.8 (11.0) | 66.5 (11.5) | **<0.001** |
| Male sex, n (%) | 210 (68.0%) | 247 (80.2%) | 249 (80.6%) | 222 (72.1%) | 928 (75.2%) | **<0.001** |
| White race, n (%) | 178 (57.6%) | 186 (60.4%) | 207 (67.0%) | 171 (55.5%) | 742 (60.1%) | **0.021** |
| Ever alcohol, n (%) | 151 (48.9%) | 150 (48.7%) | 150 (48.5%) | 145 (47.1%) | 596 (48.3%) | 0.969 |
| Ever smoker, n (%) | 96 (31.1%) | 98 (31.8%) | 120 (38.8%) | 109 (35.4%) | 423 (34.3%) | 0.156 |
| Diabetes, n (%) | 50 (16.2%) | 68 (22.1%) | 86 (27.8%) | 119 (38.6%) | 323 (26.2%) | **<0.001** |
| Hypertension, n (%) | 207 (67.0%) | 235 (76.3%) | 238 (77.0%) | 251 (81.5%) | 931 (75.5%) | **<0.001** |
| History of myocardial infarction, n (%) | 173 (56.0%) | 180 (58.4%) | 190 (61.5%) | 163 (52.9%) | 706 (57.2%) | 0.172 |
|  | | | | | | |
|  | Baseline BF% Q1 [4.4, 21.9) | Baseline BF% Q2 [22, 27.6) | Baseline BF% Q3 [27.7, 34.9) | Baseline BF% Q4 [35, 54.2] | Total | P-value |
| Age (years), mean (SD) | 65.7 (11.8) | 65.6 (11.8) | 66.0 (11.3) | 68.6 (10.8) | 66.5 (11.5) | **0.003** |
| Male sex, n (%) | 295 (95.5%) | 275 (87.9%) | 239 (78.6%) | 119 (38.6%) | 928 (75.2%) | **<0.001** |
| White race, n (%) | 201 (65.1%) | 181 (57.8%) | 183 (60.2%) | 177 (57.5%) | 742 (60.1%) | 0.191 |
| Ever alcohol, n (%) | 168 (54.4%) | 151 (48.2%) | 144 (47.4%) | 133 (43.2%) | 596 (48.3%) | **0.048** |
| Ever smoker, n (%) | 96 (31.1%) | 99 (31.6%) | 120 (39.5%) | 108 (35.1%) | 423 (34.3%) | 0.109 |
| Diabetes, n (%) | 55 (17.8%) | 59 (18.9%) | 93 (30.6%) | 116 (37.7%) | 323 (26.2%) | **<0.001** |
| Hypertension, n (%) | 211 (68.3%) | 239 (76.4%) | 229 (75.3%) | 252 (81.8%) | 931 (75.5%) | **0.002** |
| History of myocardial infarction, n (%) | 180 (58.3%) | 192 (61.3%) | 173 (56.9%) | 161 (52.3%) | 706 (57.2%) | 0.145 |
|  | | | | | | |
|  | Baseline LBM Q1 [11.5, 48.8) | Baseline LBM Q2 [48.9, 58.6) | Baseline LBM Q3 [58.7, 65.5) | Baseline LBM Q4 [65.5, 98.8] | Total | P-value |
| Age (years), mean (SD) | 70.1 (11.4) | 67.8 (11.3) | 65.2 (11.1) | 62.8 (11.0) | 66.5 (11.5) | **<0.001** |
| Male sex, n (%) | 58 (18.7%) | 263 (86.0%) | 306 (98.1%) | 301 (98.7%) | 928 (75.2%) | **<0.001** |
| White race, n (%) | 167 (53.7%) | 171 (55.9%) | 209 (67.0%) | 195 (63.9%) | 742 (60.1%) | **0.001** |
| Ever alcohol, n (%) | 118 (37.9%) | 137 (44.8%) | 179 (57.4%) | 162 (53.1%) | 596 (48.3%) | **<0.001** |
| Ever smoker, n (%) | 98 (31.5%) | 104 (33.4%) | 114 (36.5%) | 107 (35.1%) | 423 (34.3%) | 0.601 |
| Diabetes, n (%) | 84 (27.0%) | 89 (29.1%) | 78 (25.0%) | 72 (23.6%) | 323 (26.2%) | 0.439 |
| Hypertension, n (%) | 226 (72.7%) | 240 (78.4%) | 236 (75.6%) | 229 (75.1%) | 931 (75.5^% | 0.423 |
| History of myocardial infarction, n (%) | 173 (55.6%) | 176 (57.5%) | 182 (58.3%) | 175 (57.4%) | 706 (57.2%) | 0.920 |

*BF: body fat, BMI: body mass index, LBM: lean body mass. Anova was used for continuous measures and Fisher’s exact was used for categorical variables.Ranges for each quartile are noted above*

Supplemental Table 2. Incidence and median time to occurrence of long-term cardiovascular outcomes among study cohort (n=1234)

|  | Incidence, n (%) | Time to outcome (years), median (IQR) |
| --- | --- | --- |
| **All-cause mortality** | 93 (7.5%) | 4.0 (1.7, 5.6) |
| **Cardiac surgery** | 104 (8.4%) | 2.3 (1.1, 5.3) |
| **MI** | 69 (5.6%) | 2.8 (1.4, 5.3) |
| **CHF exacerbation** | 60 (4.9%) | 2.3 (1.1, 5.3) |
| **Stroke** | 8 (2.3%) | 4.0 (1.9. 5.5) |
